# Semiconducting Polymer Photodetectors with Electron and Hole Blocking Layers: High Detectivity in the Near-Infrared

**DOI:** 10.3390/s100706488

**Published:** 2010-07-01

**Authors:** Xiong Gong, Ming-Hong Tong, Sung Heum Park, Michelle Liu, Alex Jen, Alan J. Heeger

**Affiliations:** 1 Center for Polymers and Organic Solids, University of California Santa Barbara, Santa Barbara, CA 93106-5090, USA; E-Mails: minghong@physics.ucsb.edu (M.-H.T.); shpark@physics.ucsb.edu (S.H.P.); 2 Department of Materials Science and Engineering, University of Washington, Seattle, WA 98195-4330, USA; E-Mails: shiliu@u.washington.edu (M.L.); ajen@u.washington.edu (A.J.)

**Keywords:** semiconducting polymer, photodetectors, blocking layers, detectivity

## Abstract

Sensing from the ultraviolet-visible to the infrared is critical for a variety of industrial and scientific applications. Photodetectors with broad spectral response, from 300 nm to 1,100 nm, were fabricated using a narrow-band gap semiconducting polymer blended with a fullerene derivative. By using both an electron-blocking layer and a hole-blocking layer, the polymer photodetectors, operating at room temperature, exhibited calculated detectivities greater than 10^13^ cm Hz^1/2^/W over entire spectral range with linear dynamic range approximately 130 dB. The performance is comparable to or even better than Si photodetectors.

## 1. Introduction

Sensing from the ultraviolet (UV)-visible to the infrared is critical for a variety of industrial and scientific applications, including image sensing, communications, environmental monitoring, remote control, day- and night-time surveillance and chemical/biological sensing [1–3]. Today, separate sensors are fabricated from inorganic materials for different sub-bands within the UV to near-infrared (NIR) wavelength (λ) range [4]. Colloidal inorganic semiconductor quantum dots (PbS) were used to fabricate NIR-photodetectors onto gold interdigitated electrodes [5,6]. These NIR-photodetectors showed photoconductive gain and photoresponse out to 1,450 nm [6]. However, the quantum dot NIR-photodetectors were fabricated using the “in-plane” structure with electrode spacing >5 μm. As a result the required driving voltage is too high (>40 V) to be used with any commercially available thin film transistor (TFTs) arrays for read-out. These limitations significantly restrict the application of inorganic photodetectors in day- and night-time surveillance and chemical/biological sensing where high-speed and low power photodetectors are desired.

Polymer photodetectors (PPDs) have been the subject of extensive research in the past decade. PPDs offer a number of advantages: large-area detection, wide selection of materials, thin and light weight, low-cost fabrication on flexible substrates and operation at room temperature. PPDs with fast temporal-response have been reported [7–10]. In previous work [10], we reported PPDs with spectral response from 300 nm to 1,450 nm with detectivities larger than 10^13^ cm Hz^1/2^/W.

Although very small dark currents are required for high performance, there is no previous report that addresses how to minimize thermally generated dark currents from narrow-band gap semiconducting polymers. We report here PPDs comprising bulk heterojunction materials. By using electron and hole blocking layers, we have reduced the dark current by 3 orders of magnitude. As a result the detectivity is enhanced by a factor of 20.

## 2. Experiment

**Scheme 1.**
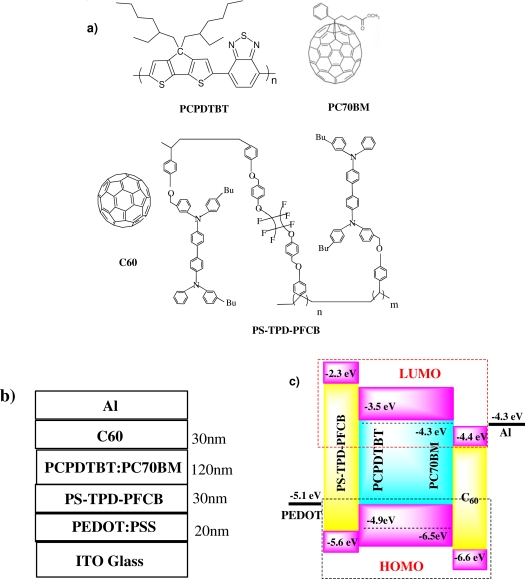
(a) Molecular structures of PCPDTBT, PC_70_BM, C_60_ and PS-TPD-PFCB; (b) Device structure; (c) Energy level diagram of PCPDTBT, PC_70_BM, C_60_, PS-TPD-PFCB, ITO and Al.

***Device Fabrication***: poly[2,6-(4,4-bis-(2-ethylhexyl)-4*H*-cyclopenta[2,1-*b*;3,4-*b*′]dithiophene)-*alt*-4,7-(2,1,3-benzothiadiazole)] (PCPDTBT) [11,12] mixed with (6,6)-phenyl-C_71_-butyric acid methyl ester (PC_70_BM) were co-dissolved in 1, 2-dichlorobenzene (ODCB) at 1:1 weight ratio and stirred overnight at 70 °C. Indium tin oxide (ITO) coated glass substrates were cleaned, sequentially, by ultrasonic treatment in detergent, de-ionized water, acetone and isopropyl alcohol, and dried overnight in an oven at >100 °C. A thin layer (∼20 nm) of poly(3,4-ethylenedioxythiophene):poly(styrenesulfonate) (PEDOT:PSS) was spin-cast onto the ITO surface. Then the PCPDTBT:PC_70_BM BHJ layer (∼120 nm) was spin-cast (1,000 rpm) from the blend solution on the modified ITO surface. For PPD A: ITO/PEDOT/PCPDTBT:PC_70_BM/Al, a ∼200 nm Al layer was thermally deposited on top of the BHJ layer and used as the top electrode. For PPD B: ITO/PEDOT/PCPDTBT:PC_70_BM/C_60_/Al, a ∼30 nm C_60_ layer was thermally deposited on top of BHJ layer, and Al (∼200 nm) was used as electrode. For PPD C: ITO/PEDOT/PS-TPD-PFCB/PCPDTBT:PC_70_BM/C_60_/Al, a ∼30 nm thin layer of polystyrene-N,N-diphenyl-N,N-bis(4-n-butylphenyl)-(1,10-biphenyl)-4,4-diamine-erfluorocyclobutane (PS-TPD-PFCB) [13] was inserted between PEDOT:PSS and active layer, by spin-casting from the corresponding solution and thermally annealed at 210 °C for 10 minutes inside the glove box. A thin layer of C_60_ (∼30 nm) was then inserted between the BHJ layer and top Al electrode. The PPD area is 4.5 mm^2^. The molecular structures of all the component materials are shown in Scheme 1a.

***Current-Voltage Measurement:*** The light source was calibrated solar simulator. For *J–V* measurement of PPDs, a band-pass filter was used to obtain the light at 800 nm. Data were collected using a Keithley 236 SMU.

***External Quantum Efficiency (EQE) Measurement:*** EQEs under short circuit was determined by illuminating the device with periodic (*i.e.,* “chopped”) monochromatic light. The AC photocurrent from the device is converted to an AC voltage and measured with a lock-in amplifier. Incident light from a xenon lamp (100 W) passing through a monochromator was chopped at 170 Hz and focused on the active area of device. A calibrated crystalline silicon diode (818UV, Newport) was used as a reference before each measurement.

## 3. Results and Discussions

**Figure 1.**
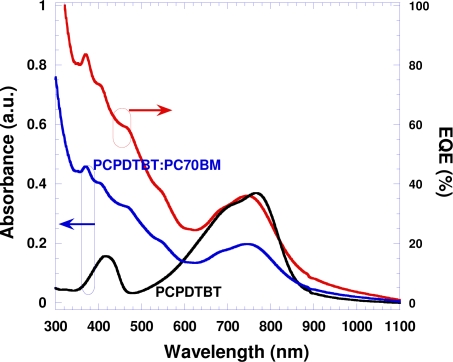
Absorption spectra (left) of pristine PCPDTBT and PCPDTBT:PC_70_BM thin films, and EQE (right) from the device with the following structure: ITO/PEDOT:PSS/PCPDTBT:PC_70_BM/Al. The EQE was measured at zero bias.

The narrow-band gap semiconducting polymer, PCPDTBT (Scheme 1a), has broad band absorption at the wavelengths λ = 300–950 nm, with a cutoff at λ ≈ 1,000 nm (Figure 1), high photoconductivity. Good solar cell performance is obtained by blending it with PC_70_BM [12].

The photo-active layer in our PPDs comprises a phase separated blend of PCPDTBT and PC_70_BM. The two components form interpenetrating donor/acceptor networks in the bulk heretojunction (BHJ) structure. Three different PPD architectures were investigated:

PPD A: ITO/PEDOT:PSS/PCPDTBT: PC_70_BM/Al;
PPD B: ITO/PEDOT:PSS/PCPDTBT: PC_70_BM/C_60_/Al and
PPD C: ITO/PEDOT:PSS/PS-TPD-PFCB/PCPDTBT: PC_70_BM/C_60_/Al.

These three device architectures are shown in Scheme 1b (the thickness of each layer is indicated). The energy level diagram in Scheme 1c shows the lowest unoccupied molecular orbital (LUMO) and the highest occupied molecular orbital (HOMO) of PCPDTBT, PC_70_BM, C_60_ and PS-TPD-PFCB. The workfunctions of PEDOT:PSS and Al are also shown in Scheme 1c. The difference between the LUMOs of PCPDTBT and PC_70_BM is ∼0.8 eV, which ensures photoinduced charge transfer and charge separation in the PCPDTBT:PC_70_BM BHJ structure [14].

**Figure 2.**
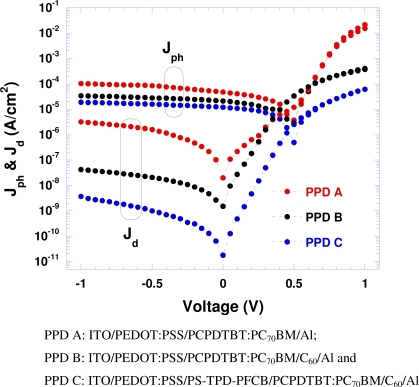
Current-density-voltage characteristics of polymer photodetectors measured in the dark (J_d_) and under light (J_ph_); λ = 800nm with intensity of 0.22 mW/cm^2^

The current-density voltage (J–V) characteristics measured in the dark and under illumination (λ = 800 nm) with light intensity of 0.22 mW/cm^2^ are shown in Figure 2. All PPDs (A, B and C) show good rectification ratios in the dark, 10^4^ at ±1 V, indicating the formation of good diodes. The dark currents observed from PPD B are more than 2 orders of magnitude smaller than that from PPD A; the dark currents observed from PPD C are more than 10 times smaller than that from PPD B. These results indicate that the thin C_60_ and PS-TPD-PFCB buffer layers are important for minimizing the dark currents generated withinfrom the PCPDTBT:PCBM PC_70_BM BHJ structure.

For PPDs A, B and C, the current density (*J*)–voltage (*V*) relationship can be described by the standard diode equation [15]:

(1) $$J=-J_{0}\left\{\text{exp}\left[\frac{q\left(V+{\mathrm{JR}}_{S}\right)}{nK_{B}T}\right]-1\right\}-\frac{V+{\mathrm{JR}}_{S}}{R_{\mathrm{SE}}}$$

where

(2) $$J_{0}=A*T^{2}\text{exp}\left(-\frac{E_{\mathrm{PF}}}{K_{B}T}\right)$$

and A* = 4πqm*K_B_^2^/*h*^3^, *J*_0_ is the saturation current density, *q* is the electron charge *V* is the voltage, *n* is the ideality factor, *K_B_* is the Boltzman constant, *T* is the absolute temperature *R_S_* is the series resistance, *R_SH_* is the shunt resistance, m* is the effective electron mass, *h* is Planck’s constant, A* is Richardson’s constant and *E_PF_* is the energy difference between the HOMO of PCPDTBT and the LUMO of PC_70_BM (∼0.6 eV).

As described above, a high dark current is expected from PPD A because *E_PF_* (∼0.6 eV) is relatively small. In PPD B, because the HOMO of C_60_ is lower than the HOMO of PCPDTBT, a thin layer of C_60_ can block holes from moving into the Al cathode, resulting in a lower dark current. In PPD C, because the LUMO of PS-TPD-PFCB is higher than the LUMO of PC_70_BM, even higher than the LUMO of PCPDTBT, a thin layer of PS-TPD-PFCB blocks electrons from moving into the ITO/PEDOT:PSS bi-layer anode. The thin layer of C_60_ also blocks holes from moving into the Al cathode. Therefore, a significantly lower dark current was observed in PPD C because of the insertion of the C_60_ hole-blocking layer and the PS-TPD-PFCB electron-blocking layer.

Moreover, due to a thin layer of C_60_ inserted between PCPDTBT:PC70BM and the Al electrode, *R_S_* in PPD B should be larger than in PPD A because *R_S_* is the sum of the contact resistance and the bulk resistance of the materials [16,17]. For PPD A, *R_S_* = R_ITO_+R_PEDOT:PSS_+R_PCPDTBT:C70BM_ For PPD B, *R_S_* = R_ITO_ + R_PEDOT:PSS_+ R_(PCPDTB:PC70BM)_ + R_C60_ + R_Al_. For PDD C, For PPD B, *R_S_* = R_ITO_ + R_PEDOT:PSS_ + R_PS-TPD-PFCB_ R_(PCPDTB:PC70BM)_ + R_C60_ + R_Al_. The *R_s_* values were obtained by fitting the *J-V* curves shown in Figure 2 to Equation 1. *R_s_* = 2.6 × 10^3^ Ω/cm^2^, 4.6 × 10^3^ Ω/cm^2^ and 2.5 × 10^4^ Ω/cm^2^ for PPD A, PPD B and PPD C, respectively. The *R_sh_* values are the following: *R_sh_* = 5.6 × 10^5^ Ω/cm^2^, 6.3 × 10^6^ Ω/cm^2^ and 4.6 × 10^8^ Ω/cm^2^ for PPD A, PPD B and PPD C, respectively. Therefore, the dark current densities in these PPDs is controlled by the blocking layers: J_D_(PPD A) > J_D_(PPD B) > J_D_(PPD C), as described by Equation (1).

In order to get photoresponsivity (*PR*), the ratio of photocurrent to incident-light power, we measured the photocurrent under the light at λ = 800 nm with a light intensity of 0.22 mW/cm^2^ as shown in Figure 2. *PR* is calculated accordingly from the observed photocurrents and the light intensity used for measurement of photo response. With a bias at 0 V, the *PR* = 217 mA/W, 96 mA/W and 54 mA/W for PPDs A, B and C, respectively. With a bias at −0.5 V, the *PR* = 387 mA/W, 129 mA/W and 72 mA/W for PPDs A, B and C, respectively. These high *PR* values demonstrate very good photoresponsivity.

We also measured the external quantum efficiency (EQE) under short-circuit and reversed bias chopping the light and using a lock-in amolifier. The data are presented in Figure 1. For comparison, the absorption spectra of pristine PCPDTBT and the composite of PCPDTBT:PC_70_BM thin films are also presented in Figure 1. The similar spectral profiles of absorption and EQE of PCPDTBT:PC_70_BM indicate that photons absorbed in IR range by both PCPDTBT and PC_70_BM contribute to the photocurrent. At λ= 800 nm, the *EQE* is 33% at 0 V and increases by a factor of 2 to 60% at −0.5 V. We note that recently several novel narrow-band gap semiconducting polymers are reported to have efficient photovoltaic activity in IR spectral region [18–21]. For example, Yao *et al.* showed spectral response extended to 1,000 nm with *EQE* of 19% at 850 nm [21]. Mühlbacher *et al.* showed 38% *EQE* around 700 nm and 13% *EQE* at 850 nm [20]. The high *EQE* observed from PCPDTBT: PC_70_BM BHJ structure imply that PPDs fabricated by PCPDTBT: PC_70_BM will exhibit high detectivity.

Assuming that the shot noise from the dark current is the dominant contribution [4,10,22], the detectivity can be expressed as

(3) $$D*=\mathrm{PR}/{\left(2qJ_{d}\right)}^{1/2}=(J_{\mathrm{ph}}/L_{\mathrm{light}})/{\left(2qJ_{d}\right)}^{1/2}$$

where *PR* is the photoresponsivity; *q* is the absolute value of electron charge (1.6 × 10^−19^ Coulombs), *J_d_* is the dark current, *J_ph_* is the photo current, and *L_light_* is the light intensity. Detectivities were calculated based on the measured photocurrent, dark current and incident light intensity (Figure 2).

Under illumination at λ = 800 nm with light intensity of 0.22 mW/cm^2^, the calculated detectivities are D* = 2.7 × 10^12^ cm Hz^1/2^/W (Jones), 4.4 × 10^12^ Jones, and 4.0 × 10^13^ Jones for PPDs A, B and C, respectively (at zero bias); D* = 5.4 × 10^11^ Jones, 1.6 × 10^12^ Jones, and 7.2 × 10^12^ Jones for PPDs A, B and C, respectively (at −0.5 V).

**Figure 3.**
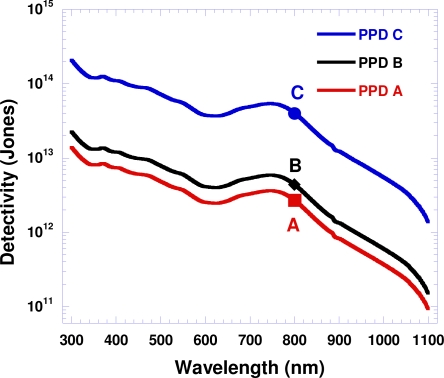
Detectivities (at 0 bias) *versus* wavelength for polymer photodetectors; the points A, B and C represent the calculated detectvities (at 0 bias) for PPDs A, B and C, respectively.

By combining the calculated detectivities at 800 nm with the photoresponsivity data, the PPDs detectivity values were obtained over the entire spectral range; the results are shown in Figure 3. The calculated detectivities at λ = 800 nm are also shown in Figure 3, represented by points A, B and C for PPDs A, B and C, respectively. Operating at room temperature, all PPDs exhibited spectral response from 300 nm to 1,100 nm. PPD C calculated showed detectivity greater than 10^13^ Jones from 300 nm to 900 nm and greater than 10^12^ Jones from 900 nm to 1,100 nm (10 times larger than observed from PPD B, and approximately 20 times larger than observed from PPD A). These results demonstrate that the electron-blocking layer, PS-TPD-PFCB, and the hole-blocking layer, C_60_, are important for achieving high detectivity NIR polymer photodetectors.

**Figure 4.**
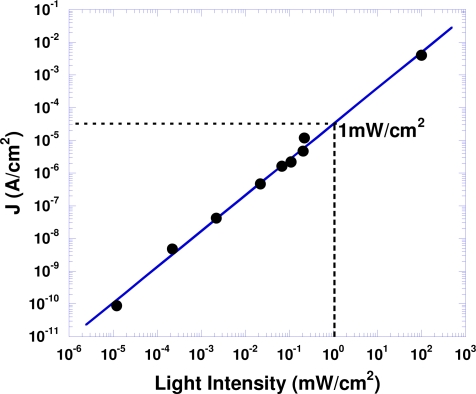
Photosensitivity *vs.* light intensity of polymer photodetector with structure ITO/PEDOT/PS-TPD-PFCB/PCPDTBT:PC_70_BM/C_60_/Al.

Figure 4 shows the photocurrent *versus* light intensity for PPD C (at λ = 800 nm). For PPD C, the photosensitivity is linear in light intensity over a range exceeding 130 dB, better than that of Si photodetectors (120 dB) [4].

In conclusion, the results presented here indicate that electron-blocking and hole-blocking layers are important for achieving high performance NIR polymer photodetectors. The results demonstrate that the performance parameters of near infrared polymer photodetectors based on PCPDTBT are comparable to or even better than Si photodetectors. The high detectivity and high photoresponsivity open opportunities for the creation of detectors with unusually wide spectral range and for the fabrication of high-resolution detector arrays for optical communications, chemical/biological sensing and day- and night-time surveillance.

## References

[1] Rogalski A, Antoszewski J, Faraone L (2009). Third-generation infrared photodetector arrays. J Appl Phys.

[2] Ettenberg M (2005). A little night vision. Adv. Imaging.

[3] Kim S, Lim YT, Soltesz E, Grand A, Lee J, Nakayama A, Parker J, Mihaljevic T, Laurence RG, Dor D, Cohn L, Bawendi M, Frangioni J (2003). Near-infrared fluorescent type II quantum dots for sentinel lymph node mapping. Nature Biotechnol.

[4] Jha AR (2000). Infrared technology.

[5] Sargent EH (2005). Infrared quantum dots. Adv. Mater.

[6] Mcdonald SA, Konstantatos G, Zhang SG, Klem EJD, Levina L, Sargent EH (2005). Solution-processed PbS quantum dot infrared photodectors and photovoltaics. Nat. Mater.

[7] Schilinsky P, Waldauf C, Brabec CJ (2002). Recombination and loss analysis in polythiophene based bulk heterojunction photodetectors. Appl. Phys. Lett.

[8] Peumans P, Bulovic V, Forrest SR (2000). Efficient, high-bandwidth organic multiplayer photodetectors. Appl. Phys. Lett.

[9] O’Brien GA, Quinn AJ, Tanner DA, Redmond GA (2006). single polymer nanowire photodetector. Adv. Mater.

[10] Gong X, Tong MH, Xia YJ, Cai WZ, Moon JS, Cao Y, Yu G, Shieh CL, Nilsson B, Heeger AJ (2009). High-detectivity polymer photodetectors with spectral response from 300 nm to 1450 nm. Science.

[11] Soci C, Hwang IW, Moses D, Zhu ZZ, Waller D, Gaudiana R, Brabec CJ, Heeger AJ (2007). Photoconductivity of a low-bandgap conjugated polymer. Adv. Func. Mater.

[12] Peet J, Kim JY, Coates NE, Ma WL, Moses D, Heeger AJ, Bazan GC (2007). Efficiency enhancement in low-bandgap polymer solar cells by processing with alkane dithiols. Nat. Mater.

[13] Gong X, Moses D, Heeger AJ, Liu S, Jen AKY (2003). High-performance polymer light-emitting diodes fabricated with a polymer hole injection. Appl. Phys. Lett.

[14] Brédas JL, Beljonne D, Coropceanu V, Cornil J (2004). Charge-transfer and energy-transfer processes in π-conjugated oligomers and polymers: a molecular picture. Chem. Rev.

[15] Nelson J (2003). The Physics of Solar Cells.

[16] Rostalski J, Meissner D (2000). Photocurrent spectroscopy for the investigation of charge carrier generation and transport mechanisms in organic p/n-junction solar cells. Sol. Energy Mater. Sol. Cells.

[17] Chang YM, Wang L, Su WF (2008). Polymer solar cells with poly(3,4-ethylenedioxythiophene) as transparent anode. Organ. Elect.

[18] Wienk MM, Turbiez MGR, Struijk MP, Fonrodona M, Janssen RAJ (2006). Low-band gap poly(di-2-thienylthienopyrazine): fullerene solar cells. Appl Phys Lett.

[19] Zhang F, Mammo W, Andersson LM, Admassie S, Andersson MR, Inganäs O (2006). Low-bandgap alternating fluorine copolymer/methanofullerene heterojunctions in efficient near-infrared polymer solar cells. Adv. Mater.

[20] Mühlbacher D, Scharber M, Morana M, Zhu Z, Waller D, Gaudiana R, Brabec CJ (2006). High photovoltaic performance of a low-bandgap polymer. Adv. Mater.

[21] Yao Y, Liang YY, Shrotriya V, Xiao SQ, Yu LP, Yang Y (2007). Plastic near-infrared photodetectors ultilizing low band gap polymer. Adv. Mater.

[22] Bhattacharya P (1997). Semiconductor Optoelectronics Device.

